# Net benefits: assessing the effectiveness of clinical networks in Australia through qualitative methods

**DOI:** 10.1186/1748-5908-7-108

**Published:** 2012-11-02

**Authors:** Frances C Cunningham, Geetha Ranmuthugala, Johanna I Westbrook, Jeffrey Braithwaite

**Affiliations:** 1 Centre for Clinical Governance Research, Australian Institute of Health Innovation, Level 1, AGSM Building, University of New South Wales, Sydney, NSW 2052, Australia; 2 Centre for Health Systems and Safety Research, Australian Institute of Health Innovation, Level 1, AGSM Building, University of New South Wales, Sydney, NSW 2052, Australia

**Keywords:** Clinical networks, Health care, Quality improvement, Health systems, Organization of care

## Abstract

**Background:**

In the 21^st^ century, government and industry are supplementing hierarchical, bureaucratic forms of organization with network forms, compatible with principles of devolved governance and decentralization of services. Clinical networks are employed as a key health policy approach to engage clinicians in improving patient care in Australia. With significant investment in such networks in Australia and internationally, it is important to assess their effectiveness and sustainability as implementation mechanisms.

**Methods:**

In two purposively selected, musculoskeletal clinical networks, members and stakeholders were interviewed to ascertain their perceptions regarding key factors relating to network effectiveness and sustainability. We adopted a three-level approach to evaluating network effectiveness: at the community, network, and member levels, across the network lifecycle.

**Results:**

Both networks studied are advisory networks displaying characteristics of the ‘enclave’ type of non-hierarchical network. They are hybrids of the mandated and natural network forms. In the short term, at member level, both networks were striving to create connectivity and collaboration of members. Over the short to medium term, at network level, both networks applied multi-disciplinary engagement in successfully developing models of care as key outputs, and disseminating information to stakeholders. In the long term, at both community and network levels, stakeholders would measure effectiveness by the broader statewide influence of the network in changing and improving practice. At community level, in the long term, stakeholders acknowledged both networks had raised the profile, and provided a ‘voice’ for musculoskeletal conditions, evidencing some progress with implementation of the network mission while pursuing additional implementation strategies.

**Conclusions:**

This research sheds light on stakeholders’ perceptions of assessing clinical network effectiveness at community, network, and member levels during the network’s timeline, and on the role of networks and their contribution. Overall, stakeholders reported positive momentum and useful progress in network growth and development, and saw their networks as providing valuable mechanisms for meeting instrumental goals and pursuing collaborative interests. Network forms can prove their utility in addressing ‘wicked problems,’ and these Australian clinical networks present a practical approach to the difficult issue of clinician engagement in state-level implementation of best practice for improving patient care and outcomes.

## Background

### Networks in health care

As a post-bureaucratic form of organization, compatible with principles of devolved governance and decentralization of services [1,2], health system ‘reform’ in many countries has looked to forms of network governance [3]. There is a considerable body of literature on networks in the non-health sector [4,5], along with research examining professional and organizational networks in the health sector [6]. The advantages of network coordination in both public and private sectors are listed by Provan and Kenis [7] as: enhanced learning, more efficient use of resources, increased capacity to plan for and address complex problems, greater competitiveness, and better services for clients and customers.

Still, Provan and Kenis identify a considerable discrepancy between the acclamation and attention networks receive and the knowledge we have about their overall functioning: that is, the process by which certain network conditions lead to various network-level outcomes is unclear. Although there is a developing literature on networks as a unit of analysis, most has been descriptive (*e.g.*, [8-10]). In the health sector too, claims for the effectiveness of networks and communities of practice tend to be theoretical or conceptual rather than empirical [11].

The term ‘network’ is used extensively in healthcare research and in health services delivery. It is often a synonym for ‘partnership,’ ‘collaboration,’ ‘alliance,’ and ‘group,’ and the term is also mobilized to describe the relationships between people, groups or organizations. The key feature of a network is the repeated, enduring exchange relationships between participating actors in the network [12]. Networks are common in the health sector: not only are they structures for service provision, but they facilitate flexible engagement at different levels throughout the health system. Network theory can be applied to a plethora of social phenomena, from individual creativity to corporate profitability [13], and social network research covers a wide range of disciplines in the social, natural, and physical sciences [14]. However, as proposed by Provan and Kenis [7], it is important to combine both network analytical and governance perspectives to examine different network governance configurations and the conditions for the effectiveness of each form.

A recent systematic review summarises the literature on the structure of health professional networks and their effectiveness and sustainability, particularly in relation to quality and safety [6]. Most of the 26 studies identified used network theory and social network analysis to examine aspects of network structure, and of structure in relation to network outcomes and sustainability. While the earlier studies in this field examined only the structural aspects of networks, more recent work examined both network structure and outputs, showing that cohesive and collaborative health professional networks can facilitate the coordination of care and contribute to improving the quality and safety of care. However, structural network vulnerabilities can include cliques, professional and gender homophily, and over-reliance on central agencies or individuals.

### International variations on the network theme

In the health system, types of networks may be seen in terms of a broad spectrum, from loose forums to share information and experiences to defined organizations and more tightly integrated forms. Quality improvement collaboratives (QICs) are one type of clinical network. Initially implemented in the United States (US) and the United Kingdom (UK), followed by adoption in many countries including Australia over the last 10 years, this type of network has attracted considerable research activity. According to Mittman [15], QICs are presented as ‘arguably the healthcare delivery industry’s most important response to quality and safety gaps’ and represent substantial investments of time, effort, and funding. Summarizing the literature on QICs, Dückers *et al.*[16] state: ‘the problem is that despite its popularity, the evidence for QIC effectiveness is positive but limited.’ They call for additional research to test the effectiveness of QICs as a spread agency, and to examine their sustainability.

The English National Health Service (NHS) funded a ‘Networks Programme’ of research on clinical networks, starting in 2004. Such research includes an NHS report summarizing lessons for network structure, management, and governance based on a literature review by Goodwin [17] and several major studies. Sheaff *et al.*[18] compared seven health networks using social network analysis and comparative case studies. They found that organizations that were more central to the network had better outcomes in terms of admissions preventable by primary-secondary coordination.

In their review of eight UK managed clinical networks, Ferlie *et al.*[19-21] employed a qualitative approach to performance assessment. Governance in this new form of healthcare organization did not fit traditional professional dominance, new public management, or market-led paradigms. These researchers engaged in Foucauldian analysis of the evidence-based medicine movement—conceived of as a ‘power-knowledge nexus’ with ‘the subjectification of clinical-managerial hybrids’ [22]. Acknowledging that ‘the ‘wicked problems problem’ remains of pervasive importance’ [19], p. 191, they conclude that ‘despite the limited progress made so far, many arguments can be found in the cases for the utility of network forms in tackling ‘wicked problems’.’ Networks provide ‘a nascent solution that needs more time to develop’ [20]. Countering concerns of post-bureaucratic critics [23,24], the researchers found that in these clinical managerial hybrids a stable professionalized leadership configuration emerged ‘as an alternative organizational core to one based on fragile general managerial roles’ [20], p. 321. Ferlie *et al.*[19] noted methodological difficulties in assessing network performance, with the need to complement qualitative data with more quantitative or even clinical outcome data, and identified the importance of developing better research methods.

Research by Guthrie *et al.*[25] on Scottish managed clinical networks found that there was no one-size-fits-all model for clinical network creators to follow, because local context, including the nature of the condition on which the network focuses, will influence what is best. More mature networks increasingly focused on relationships with their host NHS organizations, seeking to engage Health Boards and co-opt Boards’ managerial, commissioning, and contractual authority to support network goals. Currie *et al.*[26] used mixed methods (social network analysis (SNA) and qualitative fieldwork) to examine the current status and potential for leadership agency and knowledge management to transcend institutional hurdles and so ensure networks are networked. They found there was no template for the introduction of networks that was likely to fit all health and social care contexts. Contingent aspects included: concentration of professional power; the extent of externally imposed performance management; the temporal dimension of development of networks; whether network staff were co-located or not; professional work arrangements prior to implementation of networks; and local level relationships between network staff. They recommended applying generic organization studies literature (*i.e.*, developed in private sector settings) to health and social care, and building mixed methods into studies of networks.

Clinical networks are being employed as a key health policy approach to engage clinicians in improving patient care in Australia [27]. The clinical and health networks manifest predominantly as state-government facilitated, multidisciplinary, advisory groupings of health professionals and consumers, with common professional interests in particular care or services, *e.g.,* cancer, stroke, or respiratory disease. Several authors have described the development of the first Australian models of clinical networks in New South Wales (NSW) [28-30]. To re-engage clinicians in the health system, clinical networks were established in 2001 through the auspices of the Greater Metropolitan Transition Taskforce. There are now 26 statewide clinical networks, governed by the Agency for Clinical Innovation (ACI), which reports to the Director-General of the Ministry of Health and the NSW Minister for Health.

Other states have established clinical networks, usually following release of a state government report with recommendations for their establishment. In 2005, Queensland established statewide clinical networks, and there are now 12 clinical networks. Western Australia established its first networks in 2006, and there are now 17 ‘health networks,’ named to reflect the focus across the continuum of care from prevention, to palliation in all health settings. South Australia operates nine statewide clinical networks, Tasmania has five clinical networks, and the Australian Capital Territory has a chronic disease clinical network. At the national level, the Australian government has announced the investment of $58 million to establish Lead Clinicians Groups in Local Hospital Networks and a National Lead Clinicians Group to provide Ministerial advice on priorities and strategies to improve patient care, promote evidence-based clinical practices, and assist with the prioritization and implementation of clinical standards [31,32]. The local groups are expected to work closely with Local Hospital Networks, Medicare Locals (regional primary care networks) and existing state and territory clinical advisory structures.

### Aims

With a significant level of government investment in clinical and health networks in Australia and internationally, it is important to assess the effectiveness and sustainability of clinical and health networks, and the relationship of network features to effectiveness and sustainability over time. The research reported here is part of a larger study to progress the development of measures and tools for a framework to evaluate health networks and communities of practice [11]. This paper describes the features and roles of two Australian clinical/health networks and reports on the qualitative analysis of stakeholder views of the factors relating to measurement of the effectiveness of the networks. We examine the broad outputs of each network and explore the achievements of the two networks, as identified by members and stakeholders. The aim of the qualitative analysis is to assist with providing details of the context of both networks and insights into understanding the factors influencing network effectiveness from the perspective of network members and stakeholders.

## Methods

### Measuring network effectiveness

Benchmark work by Provan and Milward [33] and Provan and Sebastian [34] identified that evaluating the effectiveness of multi-firm networks was extremely complex and generally neglected. From their studies examining the links, or inter-organizational ties of mental health provider agencies, Provan and Milward [35] proposed a framework for evaluating public-sector organizational networks at three levels of analysis: community, network and organization/participant levels. Their work [36] examined network structural characteristics, and they found that the relationship between network structure and network effectiveness was mediated by network context (*e.g.,* network resources and network stability) [2]. Provan and Kenis [7] explored the impact of governance, and the role of management, on network effectiveness.

Following Provan and Milward’s contribution [35], and informed by the work of Turrini *et al.*[37] who added to their framework, we adopted a three level approach to evaluating network effectiveness: at the community level, the network level and the member level. We also recognized the three additional dimensions proposed by Turrini *et al.*[37] and applied by Ferlie *et al.*[19]: ability to reach stated goals, capacity for innovation and change, and sustainability, and viability. Also, stakeholders in both our networks recognized the temporal aspect of measuring network effectiveness and the need for appropriate measurement for the network’s development stage. Therefore, our approach addresses how evaluation can be applied at the three levels, while recognizing the three additional dimensions, across the network lifecycle. We also consider network governance as a determinant of network effectiveness.

In evaluating the network at the community level, we examine the effectiveness of the network in having an impact on services to community members. This is of importance for policy makers at the state level, and for governments who monitor and fund networks, in terms of the most efficient allocation of funds. In addition, those who represent these clients, such as consumer lobby organizations, must be satisfied by the network’s activities, in addition to the broader public. Networks can also be evaluated at the community level in terms of their contribution to building social capital [38]. The cooperation and collaboration among members engendered by the network may benefit the community in ways that would not have been possible if no social capital had been created and maintained [35].

At the network level, if the network is to be effective in achieving its objectives, the network itself must become a viable entity if it is to survive. As explained by Provan and Milward [35], to operate effectively individual members must act as a network, and this means incurring organizing and transaction costs. Some of these costs will be borne by individual members, through their voluntary membership of the network. However, because these networks are established by government bodies, network establishment and maintenance is led, coordinated, and governed by a central, local administrative entity. This entity is referred to as a network broker by Lawless and Moore [39] and Mandell [40]. As the disseminator of funds and resources, administrator, and coordinator of the network, Provan and Milward [35] contend that this broker is both the agent of the community and the principal of the network participants. One measure of network-level effectiveness is the attraction and retention of members. Network outputs also provide a further measure, along with the efficiency of information flows in the network, and the strength of relationships among members across the network. Provan and Milward [35] suggest that a further way of evaluating network effectiveness is by evaluating its administrative structure, and the role of its core coordinating and governance agency.

At the participant or member level, network affiliation may accrue benefits for the member. This may include gaining new knowledge, facilitating collaboration, professional acknowledgement, and collegiate support. Through network involvement, members may also change their own practice and effect broader practice change in their organizations.

### Study design

The study design for the evaluation of two clinical networks is based on a longitudinal comparative case study approach. This design allows individual cases to be analyzed for corroboration of specific propositions, with cross-case analysis allowing patterns to be perceived more easily and chance associations eliminated [41-43]. Our approach includes: document analysis of reports and other background documentation; the conduct of semi-structured interviews of government officials, network managers, members, and other stakeholders; the administration in November 2011 to January 2012 (time one) of an on-line survey (including sociometric questions for social network analysis) to examine network characteristics and effectiveness; analysis of the study data and feedback of initial results to the two networks; re-administration of the network survey in 2012 (time two) to examine changes to the networks over time; and data analysis and final reporting. This paper reports on the analysis of the qualitative data gathered by interviewing network managers, network members, and other stakeholders.

The document analysis of reports and other background documentation provides details of the establishment, objectives, work plans, and outputs of the networks. The direct interviews with network managers, members, and stakeholders furnish valuable first-hand insights into the governance, roles, and functioning of the networks from the perspective of network members and stakeholders. This qualitative data provides a better understanding of the context of both networks, provides confirmatory local information to operationalize core dimensions of connectivity, and identifies specific measurable variables related to working successfully in a network.

### Study setting, data collection and analysis

We purposively selected two Australian state-based clinical networks, the NSW Musculoskeletal Clinical Network and the WA Musculoskeletal Health Network. Selection of networks with a focus on musculoskeletal conditions was partly influenced by the fact that these conditions are a leading cause of long-term disability in Australia [44]. Musculoskeletal conditions need to be treated across the continuum of care, and can benefit from a multi-disciplinary health team approach, presenting complex challenges to existing care ‘silos’ and the separate federal and state, and public and private funding streams in Australia. The private sector occupies a significant role in the Australian health sector, and this is especially relevant to musculoskeletal disease, as evidenced by data from the Australian Institute of Health and Welfare [45]:. In 2010-11, almost 23% of all Australian surgical procedures reported were for ‘procedures on musculoskeletal system,’ with 81% of these occurring in private hospitals (non-profit and for-profit).

The qualitative research that we report on here was based on fieldwork, including interviews directed at ascertaining the perceptions of network members and stakeholders regarding the key factors relating to clinical network effectiveness and sustainability. Several in-depth background interviews were conducted with key informants (*e.g.,* senior department of health representatives) in each state prior to the commencement of the formal interviews in NSW and WA. Then, a total of 36 in-depth stakeholder interviews were conducted. In NSW, with 92 core members, 19 interviews (13 hours of interviews) were conducted, and in WA, with 34 core members, 17 interviews (11 hours of interviews) were conducted. In consultation with the network managers, network members were identified for interview, to provide representation of as broad a range of network members as possible. From the researchers’ background investigations, external stakeholders were also selected for interview. A snowballing technique was used where interviewees were asked if they could suggest additional key people who should be interviewed in relation to the network. Interviews continued in both states until a satisfactory breadth of representation had been achieved, and until it was clear that additional interviews were presenting no new information.

A semi-structured interview form with 46 open-ended questions was used to interview the two network managers (Additional File 1: Guide for Semi-Structured Network Manager Interviews). A shorter semi-structured interview form with 25 open-ended questions was used to interview network members (specialist clinicians, nurses, allied health practitioners, consumers, health service planners, representatives of non-government health consumer organizations) and stakeholders (representatives from the two Departments of Health and from clinical and non-government organizations). The Guide for Semi-Structured Stakeholder Interviews is available in Additional File 2. The interview schedules used a similar approach to that applied by Verburg and Andriessen [46] in their assessment of communities of practice. Probing questions were used where necessary by the interviewer for clarification (*e.g.*, to clarify whether comments on network effectiveness related to the short term, medium term or longer term), and to elucidate issues raised by the interviewees.

We collected information on key network characteristics including: establishment and objectives; governance; leadership; scope and membership; implementation and resourcing; and network outputs. All interviews were conducted face-to-face, apart from three that were conducted by telephone. Interviews took place between March and August 2011 and were all conducted by the first author. The research study procedures received ethics approval (University of New South Wales Human Research Ethics Committee Approval No. 09085) prior to recruitment and data collection. Before conducting interviews, we obtained written informed consent from all participants using an approved participant’s information and informed consent form.

All interviews were audio recorded, fully transcribed and NVivo software was used to assist with qualitative analysis of the data. First, the data were read repeatedly by the first author to achieve immersion and obtain a sense of the whole [47]. Then, text data were coded according to the specific interview questions to identify and collate respondent answers, and content analysis was employed for systematic comparison of responses across members for each network.

## Results

### Features of clinical networks

Table 1 shows the key features of the two clinical networks. Both networks have aspects of Goodwin *et al.*’s [48] ‘enclave’ type of network, with a non-hierarchical structure. Both were established directly or indirectly by state governments who have provided the network management, support and the mechanisms to bring the statewide networks together. Both networks are hybrids of the mandated and natural forms described by Braithwaite *et al.*[11]. Although established by government, network members have voluntarily joined the networks. The two networks have different governance arrangements. The NSW clinical network is governed by a separate agency, established under specific legislation, the ACI [49], while the WA health network is governed through the Health Networks Branch of the WA Department of Health. The WA structure means that the network should be more closely aligned with Departmental strategies and priorities, while still maintaining a consumer-centred focus. On the other hand, the NSW structure allows for clinical engagement, with some independence from the Ministry of Health. The different structures may have different impacts on the implementation and resourcing of policy and practice frameworks and outputs.

**Table 1:** Key features of clinical networks

**Feature**	**NSW clinical network**	**WA health network**
**Established**	2009 (developing to mature network)	2006 (mature network)
**Goal**	To advise the NSW Ministry of Health on how best to improve services for people in NSW with musculoskeletal disorders.	To provide advice and direction on where and how services should be delivered for West Australian people with musculoskeletal conditions.
**Number of members**	92 core members (170 on email list)	34 (500 on email list)
**Governance**	NSW Agency for Clinical Innovation	WA Department of Health, Networks Branch, Division of the Office of the Chief Medical Officer
**Network structure**	Network	Network, Executive Advisory Group
	Working Groups and Sub-Groups	Working Groups
**Focus**	Musculoskeletal disease	Musculoskeletal health – the right care, at the right time, in the right place, by the right team.
**Network leader**	2 specialist clinician co-chairs	Currently a network manager (Health Networks Branch, Dept. of Health); previously a Clinical Lead.
**Network management**	Network manager and 2 officers	Network manager (and Branch support)
**Membership**	Physician and surgical specialists, GPs, nurses, allied health, consumers, NGOs, researchers and academics, policy analysts, health service managers, NSW Clinical Excellence Commission, Medicare Locals, Health Education and Training Institute.	Physician and surgical specialists, GPs, allied health, nurses, consumers, carers, NGOs, Area Health Service health planners, WA Country Health Service, policy makers, researchers and academics.
**Joining network**	Contact network manager	Online registration or direct contact.
**Funding**	Through NSW Agency for Clinical Innovation, specific network funding.	Through Department of Health, in overall Branch budget.
**Working Groups**	• Osteoarthritis Chronic Care Program	• Osteoporosis Model of Care
	• Osteoporosis Refracture Prevention	• Spinal Pain Model of Care
	• Paediatric Rheumatology	• Inflammatory Arthritis Model of Care
	• Elective Joint Replacement Guideline	
	• Curriculum Development on Osteoporosis for Junior Doctors	• Elective Joint Replacement Service Model of Care
	• Musculoskeletal Nurse Graduate Certificate Development	• Others established as needed.

### Assessment of network effectiveness: community level

We examined network effectiveness at the community, network, and member levels, across network lifecycles, while considering network ability to reach stated goals, capacity for innovation and change, and sustainability. Table 2 summarizes the detailed views of interviewees on measurement of network effectiveness at the three levels, over the short, medium, and long term. At the community level, in the short term, interviewees in both states would measure effectiveness through the level of acceptance of network recommendations. This would include acceptance of network MoCs by everyone involved in musculoskeletal care. For the medium term, interviewees in both states would assess effectiveness through the extent of adoption of the MoCs and other network outputs in their state health system. Long term, interviewees in both states would measure effectiveness through changes in patient care. Interestingly, interviewees in NSW also identified the need to measure effectiveness over the long term by improvement in patient outcomes. However, this difference may simply be an artefact of the data collection method by interview, rather than a real difference between the states.

**Table 2:** Measurement of network effectiveness by NSW and WA interviewees

**Level**	**Measurement network effectiveness-NSW**	**Measurement network effectiveness-WA**
**Community**	Short term:	Short term:
	• Extent of consultation for MoC	• Acceptance of the recommendations by everyone involved in musculoskeletal care
	• Clinician agreement with MoCs	
	• Awareness of network by musculoskeletal clinicians	
		Medium term:
		• Adoption of the MoCs into the health care system
	Medium term:	
	• Demonstrated network outputs	
		• Implementation of MoCs (extent of, timeliness)
	• Awareness of MoCs by musculoskeletal clinicians	
		• Knowledge of broader clinical community of MoCs (e.g., in primary care)
	• Involvement of musculoskeletal clinicians with MoCs	
		Long term:
	• Enabling and empowerment of clinicians to contribute	
		• Changes in patient care, e.g., how referrals happen, timeliness of patient access, patient information, information feedback to general practitioners
	• Extent of implementation into hospitals of MoCs and other network outputs across NSW	
	• Adaptation of the MoC in NSW	
		• Making a difference with grassroots service providers
	• Availability of funding for MoC implementaion	
		• Alignment of care delivery with network recommendations
		• Embracing of MoCs by community
	Long term:	
	• Changing and improving practice	• Sustainability of projects
	• Improvement of patient care and services for patients	
	• Measureable difference in patient outcomes and satisfaction, attributable to the MoCs	
**Network**	Short term (getting the network together):	Short term (getting the network together):
	• Developing a collegiate network of clinicians to sustain the development of the network	• Investment in network processes from Department of Health
	• Broad representation of key stakeholders in network – e.g., across continuum of care, geographically, specialist-wise and educationally	• Broad range of stakeholders on network
		• Number of members on network
	• Involvement of best clinicians in network	• Engagement with all stakeholders
	• Egalitarian processes in network	• Happy, energetic leaders
	• Movement towards network objectives	• Continuous communication in network
	• Confidence of funding bodies in network, and their perceptions of network	• Contribution of network manager
		Medium term (getting the network functioning):
	• Clinician enablement and empowerment to contribute	• Meeting network strategic plan objectives and KPIs
	• Reaching consensus on clinical indicators or outcome measures	• Development of MoCs
	• Capacity to identify a clinical problem	• Research productivity (outputs) linked to the MoCs
	• Timeliness, availability of MoCs, level of consultation for MoCs	
		• Recognition of role of network – the visibility of the network
	• Commitment of network chairs	• Development of MoCs
			• Contribution of network manager
	Medium term (getting the network functioning):	• Research productivity (outputs) linked to the MoCs	
	• Development of many MoCs	• Recognition of role of network – the visibility of the network	
	• Capacity to identify a clinical problem	• Network outputs	
	• Timeliness, availability of MoCs, level of consultation for MoCs		
	• Commitment of network chairs		
	• Contribution of network manager	Long term (selling the plan):	
	Medium term (getting the network functioning):	• Influence on policy	
	• Development of many MoCs	• Influence on planning	
	• Capacity to implement measurable, practical, sustainable changes	• Influence on practice	
	• Focus of attention through network on musculoskeletal issues		
	• Contribution of network to development of new evidence		
	Long term (selling the plan):		
	• Getting people together will change behaviour through cultural change in the way clinicians treat musculoskeletal disease		
	• Meeting network Key Performance Indicators, e.g., reducing refractures within the network Refracture MoC.		
	• Achievement on a state-wide scale, not just for single institutions		
**Member**	Short term:	Short term:	
	• Member participation and responsiveness in the network	• Member participation and performance in network	
	• Spirit of member action on their objectives and volunteer input	Medium term:	
		• Honouring of people’s investment and time	
	Medium term:		
		Long term:	
	• Recognition by hospital/LHD of member contribution to Network		
		• Influence on practice of members	
	Long term:		
	• Embedding practice change in member’s hospital or place of work		

### Assessment of network effectiveness: network level

At the network level, over the short term, the focus was on getting the network together. Interviewees in both states would measure effectiveness by growth in network membership, by achieving broad stakeholder representation, and by the contribution of the network manager and network leadership. In NSW, with the network at an earlier stage in its lifecycle compared with WA, interviewees also noted some of the network processes that they considered important in the short term. These processes included network capacity to identify a clinical problem, egalitarian processes in the network, and clinician enablement and empowerment to contribute. Over the medium term, interviewees in both states would measure effectiveness through the development of MoCs and network outputs, by the contribution of the network to new research evidence relevant to the MoCs, and by the network’s achievement in focussing attention on musculoskeletal issues. Over the long term, interviewees in both states would measure effectiveness by the broader statewide influence of the network on practice change.

### Assessment of network effectiveness: member level

At the member level, in the short term, interviewees in both states would assess effectiveness in terms of individual member participation and performance in the network. In the medium term, interviewees in both states identified the importance of recognition of the member’s contribution to the network at the member’s workplace. Longer term, interviewees in both states would assess effectiveness through the embedding of practice changes in the member’s own hospital or place of work.

### Role of networks and their contribution

NSW stakeholders were strongly positive about the progress of their network in the short period since its mid-2009 establishment. They commented especially on network effectiveness in identifying areas of need, under-detection, or under-treatment, and developing best practice through evidence-based and patient-driven MoCs. They saw the role of the network as an interface for state-based musculoskeletal clinical expertise, bringing ‘clout’ for networking and lobbying at a governmental level. The network was viewed as a knowledge broker across the health ‘silos,’ bringing together consumer needs across the continuum of care. Stakeholders noted the role of the network in engaging clinicians and enabling clinicians to contribute to policy. They also identified the network’s role in including multidisciplinary teams, getting stakeholder buy-in, and getting all the different groups involved.

However a potential vulnerability was identified by NSW stakeholders with a disconnection between the network’s MoC recommendations and implementation, particularly where authority for implementation does not reside with the network, or the ACI, and must be done through Local Health Districts (regional hospital authorities) and their health services. A further vulnerability was identified when funding, and/or additional staffing were essential, and such approval had to be sought via the Ministry of Health. However, recently the network has worked cooperatively with the NSW Surgical Services Taskforce, NSW Ministry of Health, to implement aspects of the Osteoarthritis Chronic Care Program MoC.

NSW stakeholder suggestions to facilitate MoC implementation included: embedding the MoCs as criteria for service recognition and for funding, defining more clearly for the ACI and the Ministry of Health, the relationship and decision-making process about resourcing and distribution of scant resources on the basis of service models and MoCs, and making the network less ‘acute-centric.’ Other challenges identified were the difficulties in achieving team-working in musculoskeletal and orthopedic care, as illustrated by a network member’s comment: ‘I don’t think until we get better collegiality and teams working together it will work.’

Overall, WA stakeholders perceived that the network had made a valuable contribution and was working well with a high level of energy. They were strongly positive about: the network role in identifying gaps between current practice and evidence-based practice and offering recommendations; directing care into more evidence-based practices, in improving systems, and the improvement of the professional/patient interface; the role of the network in building collaboration, communication and networking across health sites; the network getting runs on the board by focusing on small activities, quick wins, and getting the message out; the effectiveness of the network structure regarding consultation processes for the MoCs; the engagement of a broad range of experts across the care continuum; the establishment of a dialogue with the orthopedic surgeons that did not exist before; and the inclusion of significant involvement of stakeholders and consumers.

In WA, while stakeholders supported the concept of the musculoskeletal network, concerns raised by stakeholders were: the issue of implementation (according to one member, ‘there needed to be money attached to the Models of Care to change things because you’re not changing it that much, but any change in health costs money and hospitals won’t do anything to change unless they’ve got dollars, and you have to have individuals within the services to drive it’); and the barriers between fund holders: the federal, state, and local governments. In WA, the State Health Executive Forum (SHEF) can ‘note’ a MoC for implementation. In the current tighter state budgetary framework, while additional funding may not always accompany support, it may not be necessary to implement certain MoC recommendations.

The WA Spinal MoC provides an example of the strategies the network employs in moving from MoC development to implementation. Following development of the MoC, a Spinal Implementation Working Group was established. This group developed a matrix to focus on the key recommendations, showing the stated objectives of the MoC, identifying the current gaps and directions for the future, and estimating the cost of changes, and sustainability. The group then determined key targets, with one of the early targets being education, both of healthcare professionals and patients, with inclusion of more rural and remote areas of the state. A ‘roadshow’ was conducted at four WA sites, a patient pamphlet was developed on back pain, and endorsed by different professional colleges and pain societies. In addition, information will be available on a website.

A number of WA stakeholders gave the example of the new clinical structure at South Metropolitan Area Health Service (accompanying the development of the new Fiona Stanley Hospital) as creating opportunities for implementation of MoCs through operational line management, leading patient flow, and activity and budget across the area. In WA, a number of interviewees suggested that when the Commonwealth reform of ‘activity-based funding’ was implemented in public hospitals in 2012, that the MoCs might prove useful in guiding care pathways associated with such funding, thereby giving access to implementation for the MoCs and associated funding incentives.

In both states, challenges include involving the non-acute sector, and rural, indigenous, and private representation. Both networks have endeavoured to include the non-acute sector, and there is appreciation of the challenge of involving private practitioners, *e.g.*, general practitioners or allied health practitioners, when attendance at network meetings may mean foregoing income. The involvement of rural and indigenous representation presents particular challenges in the Australian setting. Given the significant role of the private sector in the treatment of musculoskeletal disease in Australia, greater involvement of the private sector is required in these networks, from the perspective of improving outcomes for all those with musculoskeletal disease.

Achieving actual implementation of the MoCs is a litmus test of the effectiveness of clinical networks. Aligning clinical/health network work strategically with health department policy and with service level implementation are significant challenges. Where MoC recommendations cover primary care innovations, these are outside the operational and funding remit of state health departments, and are not a priority area for state resourcing.

### Key outputs and achievements of networks

The key outputs and achievements of the two networks are shown in Tables 3, 4, and 5. Both networks used a distributed leadership model, with a collaborative, inclusive style, and both have used a structure of establishing key working groups, led by expert network members with responsibility to develop specific MoCs. In both networks, the network manager and network leadership were very highly regarded and perceived by stakeholders as being critical to network effectiveness. Based on stakeholder views provided in interviews, the achievements of the two networks are summarized in Table 5, at the member, network, and community levels.

**Table 3:** Results—Key outputs of NSW clinical network

**Working Group**	**Output**
**Osteoarthritis Chronic Care Program WG**	• Osteoarthritis Chronic Care Program Model of Care (Consultation Draft – October 2011)
**Osteoporosis Refracture Prevention WG**	• Osteoporotic Refracture Prevention Model of Care (January 2011)
**Paediatric Rheumatology WG**	• Work in progress
**Elective Joint Replacement Guideline WG**	• Work in progress
**Curriculum Development on Osteoporosis for Junior Doctors WG**	• Developing intranet-based training program for junior doctors
**Musculoskeletal Nurse Education Program WG**	• Collaborating with the College of Nursing in the development of a Musculoskeletal Nursing Graduate Certificate
**Overall Network**	• Trial of NSW Osteoarthritis Chronic Care Program – funding secured and trials commenced
	• Involvement in Chronic Care Program – redesign projects
	• Interstate-Government officer network for Musculoskeletal Network Managers (WA, NSW)
	• Publications, conference presentations by network members
	• Assisted with Orthopedic Geriatric MoC Implementation
	• International Fracture Liaison connections
	• Forum for launch of Osteoporotic Refracture Prevention Model of Care (2011)
	• Currently undertaking a Formative Evaluation of the Osteoporotic Refracture Prevention MoC, including the set-up of a ‘Greenfield’ site
	• Network newsletters

**Table 4:** Results—Key outputs of WA health network

**Working Group**	**Output**
**Osteoporosis Model of Care WG** (now Implementation WG)	• Osteoporosis Model of Care (2011)
**Spinal Model of Care WG** (now Implementation WG)	• Spinal Pain Model of Care (2009)
	• Rural Roadshow (Kununurra, Albany, Kalgoorlie, Broome)
	• Consumer Guide to Management of Low Back Pain
	• Measurement of beliefs and likely practice behaviours in the context of back pain among emerging health professionals
**Inflammatory Arthritis WG** (now Implementation WG)	• Inflammatory Arthritis Model of Care (2009)
	• Securing funding for a University Chair of Rheumatology and Musculoskeletal Medicine
	• Allied Health Skillset for Inflammatory Arthritis
	• Study of cost-effectiveness of rheumatology service models: negotiated rheumatology services outside tertiary hospitals from 2012.
**Elective Joint Replacement Service WG** (now Implementation WG)	• Elective Joint Replacement Service Model of Care (November 2010)
**Network (overall)**	• Musculoskeletal Health Network Stakeholder Forum 2011
	• Manual Handling Guide for Carers
	• Interstate-Government officer network for Musculoskeletal Network Managers (WA, NSW)
	• Conference presentations, research grants, academic papers
	• Trial of Spinal MoC showed reductions in waiting list
	• Link with Armadale Hospital to develop new services involving a multidisciplinary team on rheumatological care
	• A number of osteoporosis projects are to be undertaken in collaboration with Osteoporosis Australia

**Table 5:** Clinical network achievements

**Network level**	**Achievements-NSW network**	**Achievements-WA network**
**Community**	• built a community of clinicians and consumers	• built a community of clinicians and consumers
	• raised profile of musculoskeletal conditions	• raised profile of musculoskeletal conditions
	• provided a ‘voice’ for musculoskeletal conditions	• provided a ‘voice’ for musculoskeletal conditions
		• progress on implementation of MoC’s recommendations
		• education of health professionals on back care management.
**Network**	• achieved multi-disciplinary clinician engagement (also engaged with the Orthopedic Association)	• achieved multi-disciplinary clinician engagement (also engaged with the Orthopedic Association)
	• provided respected source of knowledge	• provided respected source of knowledge
		• supported collaborative development of Models of Care
	• supported collaborative development of Models of Care	
		• achieved membership growth
		• disseminated information
**Member**	• improved connectivity, and collaboration of members	• improved connectivity, and collaboration of members
	• created research collaboration	• created research collaboration and involvement in WA State Health Research Grants
	• shared knowledge	
	• enhanced legitimacy	• shared knowledge
	• time cost	• enhanced legitimacy
	• conference presentations by members and publications	• time cost
		• conference presentations by members and publications

At the member level, both networks have improved the connectivity and collaboration of network members. There was substantial evidence of involvement of network members in research collaboration, additional to their network activities. At the network level, both networks succeeded in achieving multi-disciplinary clinician engagement. As a ‘health’ network, rather than a ‘clinical’ network, as reflected in the name, the WA network appears to have a broader scope on musculoskeletal health than the NSW network, *e.g.,* its membership includes chiropractic representation.

At the community level, interviewees perceived that both networks had not only raised the profile, but provided a ‘voice’ for musculoskeletal conditions. The establishment of these multidisciplinary networks to engage clinicians in improving practice and outcomes shows potential as a vehicle for addressing complex ‘wicked problems’ in healthcare. Because the WA network has been operational from 2006, it has made further progress with developing a number of MoCs and with progress on aspects of implementation of the MoCs’ recommendations compared with the NSW network, which has only been operational since mid-2009. Nevertheless, interviewees commented favorably on the significant progress that the NSW network has made in a short period of time.

## Discussion

This research sheds light on stakeholder perceptions of how clinical network effectiveness should be assessed at different stages in the network’s timeline, on the role of networks and their contribution, and on stakeholder perceptions of the key outputs and achievements of the networks. The research on the experience with Australian clinical networks adds to the international literature on clinical and health networks. According to the stakeholders, these networks are achieving some of the advantages identified by Provan and Kenis [7]—they bring together and engage clinicians and thereby provide increased capacity to plan for and address complex problems.

Although the two networks in our study are primarily advisory clinical networks and differ from the NHS managed clinical networks, there appear to be similar trends in our findings to the key findings reported from the NHS ‘Networks Program’ of research on clinical networks [3,17,19,25,26]. Consistent with the identification by Ferlie *et al.*[19] of the utility of network forms in tackling ‘wicked problems,’ these Australian disease-related networks present a practical approach to the difficult issue of clinician engagement in state-level implementation of best practice for improving patient care and outcomes. While noting the arguments from both Guthrie *et al.*[25] and Currie *et al.*[26] to the effect that there was no template for the introduction of networks that was likely to fit all local health and social care contexts, there do appear to be more consistent similarities between these Australian clinical and health networks.

In light of the advisory nature of these Australian clinical and health networks, it will be important, as identified by Guthrie *et al.*[25] in their findings on Scottish managed clinical networks, for these networks to be strategic in focusing on their relationships with their host organizations, and to seek to engage their Health Departments, Local Health Districts/Local Hospital Networks and Medicare Locals, and others in the health system, including the private sector, to gain support with implementation from their managerial and contractual authority to support network goals.

A study limitation is that this research has examined primarily qualitative interview data on the clinical/health networks. Analogous to the methodological issues that apply in the evaluation of QICs, such issues apply in the evaluation of clinical and health networks. As argued recently in relation to QIC research [50], rigorous evaluation of quality improvement efforts should include not only qualitative data but also quantitative data to assess the efficacy and sustainability of outcomes over time. Our continuing research on clinical and health networks includes quantitative analysis of network survey data. However, further work should be conducted to rigorously assess how well network initiatives have been implemented in practice, and to investigate what organizational characteristics support or hinder sustained improvements.

## Conclusion

The two study networks exhibited aspects of the ‘enclave’ type of network, with a non-hierarchical structure, and both government-established networks were hybrids of the mandated and natural forms. According to interviewees, in the long term, at the community level and at the network level, network effectiveness should be measured in terms of changes in patient care. In the long term, at the member level, interviewees would assess network effectiveness in terms of embedding practice changes in the member’s own hospital or place of work. Interviewees were strongly positive about the progress towards stated goals of both networks since establishment. As advisory networks, there are particular challenges with implementation of network MoCs and recommendations, but the networks are developing strategies to address these challenges. Overall, we found in these examples positive momentum and useful progress in network growth, development, and output. According to our participants, their networks were valuable mechanisms for meeting instrumental goals and pursuing collaborative interests.

## Abbreviations

ACI: Agency for Clinical Innovation; GP: General practitioner; LHD: Local Health District; MoC: Model of care; NHS: National Health Service; NSW: New South Wales; NGOs: Non-government organizations; QIC: Quality improvement collaborative; SNA: Social network analysis; SHEC: State Health Executive Forum; UK: United Kingdom; US: United States; WA: Western Australia; WG: Working group.

## Competing interests

Jeffrey Braithwaite is a member of the Editorial Board of Implementation Science. All authors declare that they have no competing interests.

## Authors’ contributions

FCC was primarily responsible for study coordination, data collection, analysis and interpretation, and the initial draft of the manuscript. All authors contributed to the ideas in this paper: they provided input into the study design and critiqued the data analysis and interpretation and synopsis of findings. All authors provided editorial contributions and read and approved the final manuscript.

## Supplementary Material

Additional file 1 **Guide for Semi-Structured Network Manager Interviews.** PDF file of the instrument used for the network manager interviews. (PDF 384 kb) Click here for file

Additional file 2 **Guide for Semi-Structured Stakeholder Interviews.** PDF file of the instrument used for the member and stakeholder interviews. (PDF 251 kb) Click here for file
