# Supplementary material for: Net benefits: assessing the effectiveness of clinical networks in Australia through qualitative methods
Source: Implement Sci. 2012 Nov 2;7:108. doi: 10.1186/1748-5908-7-108 (PMC3541150; doi:10.1186/1748-5908-7-108)
Supplement: Additional file 1 — Guide for Semi-Structured Network Manager Interviews. PDF file of the instrument used for the network manager interviews. (PDF 384 kb) [file 1748-5908-7-108-S1.pdf]

Centre for Clinical Governance Research, University of New South Wales

Evaluating Clinical and Health Networks using Social Network Methods

Guide for Semi-Structured Network Manager Interviews

A brief introduction will be provided to the network manager. Questions may not necessarily be asked in the order below. Interviewees may be prompted to provide further explanations to questions. Informed Consent will be requested, and permission will be requested for audio-recording as back-up for the interview.

Name of network manager:

Network sponsoring organisation:

Date of interview:

Network manager and sponsoring organisation

1. Could you describe your role, and your relationship to clinical / health network?

2. How long have you been in your current position?

Network establishment, purpose and structure

3. When was the network established?

4. Network stage: From your perspective, at what stage is the Network in terms of its life-cycle? (Check – e.g., start-up, development, mature, in decline)*

5. Membership:
    a. What are the eligibility criteria for becoming a member?
    b. How are members appointed?* (e.g., application, nomination, invitation, other)
    c. Does the clinical/health network have appropriate representation in its membership?
    d. Should anyone else be represented in the Network?[#]

6. Why was this network established? What is the history behind the network establishment?

7. Terms of Reference
    a. How were the Terms of Reference (topics) for the network selected?*
    b. What are the Terms of Reference for the network?
    c. Have these changed since the start of the network?
    d. Is there a process for updating them?

8. Could you describe the governance, representation and reporting lines of this network?

9. Could you provide a copy of the agenda and minutes of the formal network meetings for the last year, including member attendance?

Decision-making, implementing change and leadership

10. How were the specific network projects identified and developed?*

11. What sources has the network used for the evidence-base, e.g., for the Model of Care?

12. Is there a Work Plan and Key Results areas for the network? If so, which of these have been completed? (Is there a document that can be sent to the researchers?)

13. Network leadership:
    a. Who are the clinical leaders of the network?*
    b. In your view does the network have effective leadership?
    c. Could you please describe the leadership style and any strengths or weaknesses?

14. Do the clinical leaders on the musculoskeletal network contribute their time on a voluntary basis, or are they funded, and if so, by whom?

15. Should the clinical leaders also be funded for their time to focus on engaging colleagues in implementing the network strategies (e.g., the Model of Care)?

16. Was there any leadership training / professional development for the clinical leaders?

17. Has the network established Working Groups, or Sub-Groups? What are their titles, objectives, leadership, and project timelines?

18. How is change implemented?
    a. For example, is there any teaching of change skills?
    b. Do you have examples where the network is responsible for implementation of change, or where implementation is the responsibility of the Department of Health, or another body?

19. How do the decision-making processes in the network relate to those of the sponsor (Department of Health)?[#]

Network resources

20. How many direct staff support the network? What are their positions and levels?

21. How is the clinical/health network funded? (E.g., included in Agency/Department budget)

22. What are the levels of funding per annum for the network? Overall?

23. Is there senior management support for the network? (Please provide examples.)

24. What resources are provided through the network? For ongoing items, tick daily.) (Please provide examples of other resources.)

Activity	How often? (please indicate frequency in relevant column below							
	Daily	Weekly	Fortnightly	Monthly	Quarterly	Six-monthly	Annually	Less often	Never
Management support	☐	☐	☐	☐	☐	☐	☐	☐	☐
Technical support	☐	☐	☐	☐	☐	☐	☐	☐	☐
Administrative support	☐	☐	☐	☐	☐	☐	☐	☐	☐
Research support	☐	☐	☐	☐	☐	☐	☐	☐	☐
Musculoskeletal data system support	☐	☐	☐	☐	☐	☐	☐	☐	☐
Face-to-face meetings	☐	☐	☐	☐	☐	☐	☐	☐	☐
Face-to-face seminars	☐	☐	☐	☐	☐	☐	☐	☐	☐
Virtual meetings	☐	☐	☐	☐	☐	☐	☐	☐	☐
Virtual seminars (Webinars)	☐	☐	☐	☐	☐	☐	☐	☐	☐
Education/ Technical training courses delivered face-to-face	☐	☐	☐	☐	☐	☐	☐	☐	☐
Podcasted training sessions	☐	☐	☐	☐	☐	☐	☐	☐	☐
Teleconferences	☐	☐	☐	☐	☐	☐	☐	☐	☐
Website	☐	☐	☐	☐	☐	☐	☐	☐	☐
Blog	☐	☐	☐	☐	☐	☐	☐	☐	☐
Emails	☐	☐	☐	☐	☐	☐	☐	☐	☐
Newsletter	☐	☐	☐	☐	☐	☐	☐	☐	☐
Social events	☐	☐	☐	☐	☐	☐	☐	☐	☐
Other (specify)	☐	☐	☐	☐	☐	☐	☐	☐	☐
Other (specify)	☐	☐	☐	☐	☐	☐	☐	☐	☐

Network outputs / outcomes

25. For the major events hosted by the network how many people attended each? From what sectors?

26. What are some of the major outcomes and achievements of this network?

27. How does the network benefit: members as individuals, their organisation, their community level?

Network strategies

28. What sorts of strategies has the network used to achieve its goals?*(eg, developing a model of care, pathways of care, conferences, seminars, web-seminars, workshops, lobbying, other?) Please provide examples.

29. What sorts of strategies has the network used to achieve practice change?* ("Practice" is defined as the carrying out of work as it relates to one's professional role.)

30. Other objectives – what sorts of strategies has the network used to achieve these objectives?*

Barriers and facilitators to achieving outcomes

31. What are the factors that have helped the network to achieve its objectives?

32. Are you aware of any barriers to the success of this network (e.g, funding, resources, authority, legislation, regulation, culture, attitudes, etc.)

Network relationships

33. How would you describe the relationship between the clinical/health network and the Health Department?
    a. What is the role of the Health Department? (e.g., convenor, facilitator, funder).
    b. What should be the role of the Health Department?

34. How would you describe the relationship between the clinical/health network and the Local Health Networks/Clusters?

35. What linkages are there between the network and other bodies like the Department of Health, Local Health Networks/Clusters, Clinical Excellence Centre, NHMRC.

36. To whom does the network disseminate information?

Network effectiveness

37. In looking at what makes an effective clinical/health network, what factors would you use to gauge the success of this network?

38. Could you suggest ways of improving the effectiveness of the network?

39. Would you say there is 'clinician-buy in' to the network? (Please provide examples.)

40. How would you (as network manager) judge the degree of success of the network?*

41. Has the network been effective in disseminating information?

Evaluation data and monitoring

42. Have you collected baseline and trend data on incidence, prevalence of musculoskeletal disease, or waiting times / access to joint surgery?
    a. Could you please provide a copy of these datasets?
    b. Are formal reports required?
    c. Is there public reporting of results?

43. Has the network led to improved patient care (are there any data to support this)?[#]
    a. Clinical outcomes?
    b. Equity?
    c. Continuity of patient care?
    d. Knowledge sharing and innovation?
    e. Access?
    f. Speed of care?
    g. Other?

Network duration

44. What is the envisaged duration of the network? What will happen when the network has done its job?

Member survey

45. Member survey: Could you please provide a list of Network Members, with their position title, and their telephone and email contact details?

Conclusion

46. Are there any other comments you would like to make?

* Based on (Wilson et al., 2003), # based on (Ferlie et al., 2010)

References

Ferlie, E., Fitzgerald, L., McGivern, G., Dopson, S., & Exworthy, M. (2010). Networks in Health Care: A Comparative Study of their Management, Impact and Performance. Report for the National Institute for Health Research Service Delivery and Organisation Programme. SDO Project (08/1518/102). London: Department of Management, Kings College London.

Wilson, T., Berwick, D. M., & Cleary, P. D. (2003). What do collaborative improvement projects do? Experience from seven countries. *Joint Commission Journal on Quality and Safety,* 29(2), 85-93.
