# Supplementary material for: Net benefits: assessing the effectiveness of clinical networks in Australia through qualitative methods
Source: Implement Sci. 2012 Nov 2;7:108. doi: 10.1186/1748-5908-7-108 (PMC3541150; doi:10.1186/1748-5908-7-108)
Supplement: Additional file 2 — Guide for Semi-Structured Stakeholder Interviews. PDF file of the instrument used for the member and stakeholder interviews. (PDF 251 kb) [file 1748-5908-7-108-S2.pdf]

Centre for Clinical Governance Research, University of New South Wales

Evaluating Clinical and Health Networks using Social Network Methods

Guide for Semi-Structured Stakeholder Interviews

A brief introduction will be provided to the stakeholder. Questions may not necessarily be asked in the order below. Interviewees may be prompted to provide further explanations to questions. Informed Consent will be requested, and permission will be requested for audio-recording as back-up for the interview.

Name of stakeholder:

Organisation and position:

Date of interview:

Personal Role

1. Role of stakeholder, and any relationship to clinical/health network.

2. Length of time in current position.

Network establishment, purpose and structure

3. How did you find out about the network and its work?

4. Do you know any members in the Network? Who?*

5. Are you a member of any other Networks? If so, which one/s?

6. What is your perception of the network's role or contribution to the issues on which it is working?*

7. Does the clinical/health network have appropriate representation in its membership? Should anyone else be represented in the Network?#

Decision-making, implementing change and leadership

8. In your view does the network have effective leadership? Could you please describe this leadership style and any strengths or weaknesses?

9. Could you comment on the role of the network manager, in relation to the effectiveness of the network?

10. Has the network been effective in disseminating information?

11. How would you describe the relationship between the clinical/health network and the Health Department? What is the role of the Health Department? (e.g., convenor, facilitator, funder). What should be the role of the Health Department?

12. How would you describe the relationship between the clinical/health network and the Local Health Networks/Clusters?

Network achievements

13. Could you indicate some of the outcomes and achievements of this network that stand out? (prompt for model of care, workshops, conference presentations, research reports, website, newsletter, etc.) *

14. What sorts of mechanisms has the network used to achieve these outcomes (e.g., developing a model of care, pathways of care, education/training, lobbying)

15. Are the network outputs timely and of immediate importance to its constituents?*

16. Were the outputs from the Network in a format useful for you / your organisation?*

17. Is the content of the Network output material credible, reliable?*

18. Is the network recognised as an expert in musculoskeletal disease?*

19. Where is the network positioned vis-à-vis other actors in this field?

20. How does the network adopt international best practice?

Success: facilitators and barriers

21. What are the factors that have helped the network to achieve its objectives?

22. Are you aware of any barriers to the success of this network (e.g, funding, resources, authority, etc.)

23. In looking at what makes an effective clinical/health network, what factors would you use to gauge the success of this network?

24. Could you suggest ways of improving the effectiveness of the network?

25. Do you have any further comments?

* Based on (Creech & Ramji, 2004), # based on (Ferlie et al., 2010)

References

Creech, H., & Ramji, A. (2004). Knowledge networks: Guidelines for assessment. Working Paper. Winnipeg: International Institute for Sustainable Development,.

Ferlie, E., Fitzgerald, L., McGivern, G., Dopson, S., & Exworthy, M. (2010). Networks in Health Care: A Comparative Study of their Management, Impact and Performance. Report for the National Institute for Health Research Service Delivery and Organisation Programme. SDO Project (08/1518/102). London: Department of Management, Kings College London.
